# Stress phase angle regulates differentiation of human adipose-derived stem cells toward endothelial phenotype

**DOI:** 10.1007/s40204-018-0090-5

**Published:** 2018-05-21

**Authors:** Shahrokh Shojaei, Mohammad Tafazzoli-Shadpour, Mohammad Ali Shokrgozar, Nooshin Haghighipour, Fatemeh Hejazi Jahromi

**Affiliations:** 1 Faculty of Biomedical Engineering, Central Tehran Branch, Islamic Azad University, 13185/768, Tehran, Iran; 2 Cardiovascular Engineering Lab., Faculty of Biomedical Engineering, Amirkabir University of Technology, Tehran, 158754413 Iran; 3 National Cell Bank of Iran, Pasteur Institute of Iran, Tehran, 1316943551 Iran; 4 Hard Tissue Engineering Research Center, Tissue Engineering and Regenerative Medicine Institute, Central Tehran Branch, Islamic Azad University, 13185/768, Tehran, Iran

**Keywords:** Stress phase angel, Shear stress, Cyclic stretch, Adipose mesenchymal stem cells, Cell behavior

## Abstract

Endothelial cells are subjected to cyclic shear by pulsatile blood flow and pressures due to circumferential stresses. Although most of the researches on this topic have considered the effects of these two biomechanical forces separately or concurrently, few studies have noticed the interaction of these cyclic loadings on endothelial behavior. Negative temporal stress phase angle, defined by the phase lag between cyclic shear and tensile stresses, is an established parameter which is known to have substantial effects on blood vessel remodeling and progression of some serious cardiovascular diseases. In this research, intermittent shear and tensile stresses with different stress phase angle values were applied on human adipose stem cells (ASC). The expression level of three major endothelial-specific genes, elastic modulus of cells and cytoskeleton actin structure of cells were studied and compared among control and three test groups subjected to stress phase angle values at 0°, − 45°, and − 90°. Mechanical properties of ASCs were determined by atomic force microscopy and actin fiber structure was visualized by confocal imaging through Phalloidin staining. Results described a decrease in expression of FLK-1 and VE-cadherin and rise of vWF marker expression in case of higher negative stress phase angles. The Young’s moduli of cells were significantly higher and cytoskeletal actin structure was more organized with higher thickness for all test samples subjected to combined stresses; however, these features were less magnificent for applied stress phase angles with higher negative values. The results confirmed significant effects of SPA on endothelial differentiation of mesenchymal stem cells.

## Introduction

Endothelial cells (ECs) cover the entire cardiovascular bed and role as the blood–vessel wall interface within arteries. ECs are exposed to circumferential stress which is caused by pressure pulse acting on elastic vessel walls. Shear stress is another vital biomechanical stimulus applied on ECs due to the pulsatile blood flow. There is sufficient in vitro evidence that may imply to possible influence these two mechanical stresses have on many essential traits of both ECs and stem cells. For instance, it was demonstrated that shear stress affects gene expression (Paul et al. 2018), morphology and adhesion (Charrier et al. 2018; Paim et al. 2018), extracellular matrix secretion, mechanical properties, and angiogenesis ability (Charoenpanich et al. 2017; La and Tranquillo 2018; Vining and Mooney 2017) of ECs and stem cells. Some of the most significant biological characteristics of ECs can be regulated by application of pulsatile shear stress (Dolan et al. 2011); shear stress out of biological range values contribute to conditions in which vascular diseases develop such as activation of IGF-1 (Elhadj et al. 2003), uptake of LDL (Kang et al. 2014), and pathogenic remodeling (Dolan et al. 2011). Cyclic tension has also been indicated to be influential on endothelial behavior (Barron et al. 2007; Haghighipour et al. 2010). Cyclic stretch promotes smooth muscle genes in stem cells, while it attenuates endothelial specific genes among ECs (Haga et al. 2007). In addition, the substrate’s characteristics have distinct influence on morphology, mechanical properties, and other important characteristics of the cultured cells (Jeon et al. 2014).

Although these biomechanical stimuli are essential in regulation of vital biological characteristics of ECs, the interaction between these biomechanical forces has attracted less attention. There are some clues which illustrate well the dominant effects of interaction between tensile and shear stresses on endothelial behavior. While many earlier studies have stressed the influence of altered pattern of shear stress on atherogenesis (Bassiouny et al. 1992; Ku et al. 1985), some other investigations have challenged the sole role of shear stress pattern in situations such as intimal thickening in the right coronary (Joshi et al. 2004) and carotid bifurcation (Steinman et al. 2002). In general, the interaction between shear and tensile stresses has been widely suggested to play an important role on endothelial behavior (Joshi et al. 2004; Steinman et al. 2002).

The phase lag between wall shear stress (WSS) and circumferential stress (CS) within arteries, known as the stress phase angle (SPA), is a primary parameter that characterizes the interaction between these two stresses. This factor could be derived based on the phase difference between first harmonics of WSS and CS waves. It has been shown that large negative values of SPA are correlated with the regions which are vulnerable to endothelial injury and plaque formation (Tada and Tarbell 2005). Further investigations have suggested that large negative values of SPA strengthen the risk of atherosclerosis and intimal hyperplasia (Dancu and Tarbell 2006; Qiu and Tarbell 2000b). It has been documented that large negative SPA attenuates the expression of some vasodilator factors including nitric oxide (NO) and prostacyclin (Dancu and Tarbell 2006) and inhibits the production of some vasoconstrictor agents such as endothelin-1 (Qiu and Tarbell 2000a).

Since mature endothelial cells within the cardiovasculature are continuously exposed to concurrent shear and tensile forces, it has been suggested that application of such mechanical stimuli may enhance differentiation of mesenchymal stem cells toward functional endothelial phenotype (Maul et al. 2011). Previously, we reported that applying shear stress alone or combined with cyclic stretch on adipose derived stem cells (ASCs) could lead to enhanced expression of endothelial-specific genes and some other endothelial vital traits in ASCs, while the cyclic stretch alone did not improve those expressions (Shoajei et al. 2014). A same trend in elevation of expression of endothelial genes has been reported when endothelial cells are exposed to different patterns of shear stress (Yamamoto et al. 2003).

On the other hand, reduction of endothelial genes was observed when endothelial cells were subjected to cyclic stretch alone, whereas smooth muscle cell markers were intensified; such variation in gene expression is pathologically regarded as inauguration of atherosclerosis (Cevallos et al. 2006). Effects of concurrent shear and tensile loadings on endothelial and mesenchymal stem cell behavior have also been investigated (Owatverot et al. 2005; Shoajei et al. 2014). In our previous research works, we addressed the effect of in-phase (SPA = 0) shear stress and tensile stretch on gene expression, mechanical properties of the membrane, angiogenesis ability, and other vital characteristics of ASCs and endometrial stem cells (Shojaei et al. 2013a, b, c, 2015). Although there are relatively high number of studies on concurrent shear stress and tensile stretch influencing the differentiation and transdifferentiation of stem cells, the effects of interaction between cyclic shear and tensile stresses are not assessed widely and perfectly on endothelial differentiation of stem cells. To our best knowledge, this is the first time that the effects of the SPA have been investigated on gene expression, mechanical properties, and actin fibers’ organization and arrangement. Here, we hypothesized that SPA, as an indicator of such interaction, is effective in differentiation of mesenchymal stem cells toward endothelial fate.

## Materials and methods

The SPA was applied by the phase lag between two cyclic stresses with harmonic profiles. Cells without any treatment were set as control samples, while they were cultured in the same conditions as the test samples. Samples from three test groups with different SPA values were exposed to mechanical loading using custom-made apparatus, and at least three tests were carried out for each test group. Expression of endothelial specific genes, cell mechanical properties, and cytoskeletal structure was analyzed to evaluate the influence of SPA on differentiation toward endothelial phenotype. The mean ± SD values of elastic modulus of cells and gene expression levels of three endothelial specific genes were calculated for control and test groups. Statistical *t* test paired analysis was performed between control group and each test group to investigate differences among gene expression levels and elastic moduli of samples and the significant difference was set as *P* < 0.05. In addition, multifactorial ANOVA analysis was carried out with the same significant difference to compare all the tests and control groups.

### Cell preparation

ASCs were extracted according to published protocols with some minor modifications (Estes et al. 2010). Pre-umbilical fat tissues were provided from healthy female donors between 30 and 35 years, during abdominal surgery with informed consent. Fat tissues were cut into small pieces and transferred to collagenase type I solution of 0.1 g collagenase (invitrogen, USA) in 100 mL phosphate buffer saline (Sigma, USA). The solution was incubated at 37 °C, 5% CO_2,_ and 95% relative humidity for 45 min, while it was shaken every 5 min, followed by removal of undigested parts. The culture medium included Dulbecco’s modified Eagle’s medium (DMEM, Gibco, USA) with 15% fetal bovine serum (Gibco, USA). The solution was centrifuged for 5 min at 2000 rpm. Then, the supernatant was removed and the bottom pellet containing ASCs and red blood cells were dissolved in fresh culture medium. The resultant solutions were transferred to a cell culture flask (T-25) and after an overnight RBCs were easily detached by light pipetting.

### Cell characterization

The cells were characterized using flow cytometry assay by assessing two specific markers for mesenchymal stem cells (CD44, CD90) and one for hematopoietic stem cells (CD45). Briefly, 35 × 10^3^ cells were counted and suspended in micro-centrifuge tube containing 90 µL serum-PBS (5% FBS in PBS). Then, 10 µL antibody/isotype was added and the tube was shaken for 1 h while protecting from light. The micro-tube was centrifuged (800 rcf, 5 min) and the supernatant was removed; then, the pellet was resuspended in serum-PBS (500 µL). After three times washing, the flow cytometry assay was conducted by forward scatter and side-scatter analyses.

### Multipotential differentiation ability

Multipotential differentiation ability of ASCs was analyzed through adipogenic and osteogenic differentiations. Cells from passage 3 were treated by adipogenic medium containing DMEM, 10% FBS, 0.5 μM IBMX (3-isobutyl-1-methylxanthine), 1 μM dexamethasone, 10 μg/mL insulin, and 100 μM indomethacin Sigma, (USA) for 3 weeks. The medium was changed every 3 days; after then, the cells were fixed by 4% paraformaldehyde (Sigma, USA) and washed in 50% isopropanol. Lipid vacuoles were visible by oil red staining. For osteogenic differentiation, ASCs were exposed to osteogenic differentiating medium (DMEM, 10% FBS, 100 nM dexamethasone, 10 mM beta-glycerol phosphate and l-ascorbic acid 2-phosphate, Sigma, USA) for 3 weeks during which the culture medium was replaced every 3 days. Cells were then fixed by 4% formalin (Sigma, USA) and washed by PBS. Osteocyte cell matrix was visible by alizarin red (40 mM).

### Application of mechanical stimuli

ASCs were exposed to cyclic shear stress (0–2.5 dyn/cm^2^, 1 Hz) and cyclic stretch (0–10%, 1 Hz) for 24 h (Cevallos et al. 2006; Obi et al. 2012), while the SPA value was altered in three magnitudes of 0°, − 45° and − 90°. Such ranges of cyclic stretch and shear stress have been used among in vitro studies to simulate biological conditions (James et al. 1995; Nagel et al. 1994; Peng et al. 2000).

Cells were cultured on collagen coated silicon rubber membrane (PDMS) for 24 h and test samples were subjected to concurrent shear and tensile stresses with differing SPA values within the culture medium (15% fetal bovine serum [FBS-(Gibco, USA)] low-glucose Dulbecco’s modified Eagle’s medium [DMEM-(Gibco, USA)] utilizing a custom made bioreactor. The control samples were cultured with the same conditions and kept for the same duration. After treatment, cells were transferred for further analyses.

**Fig. 1:**
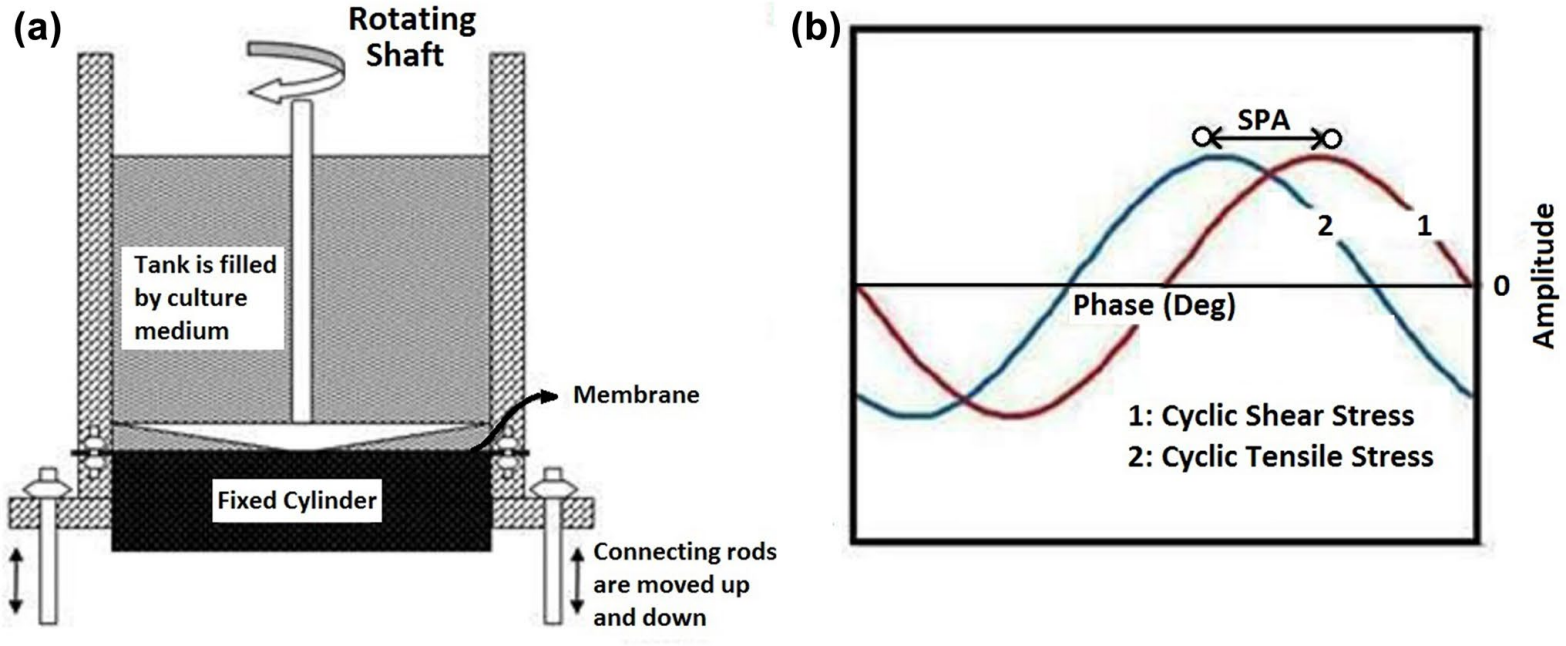
**a** Schematic view of the bioreactor. Cells are cultured on a silicone membrane and a rheometer-like mechanism including a cone and plate system has been used to create shear stress. Cone has a slight angle which guarantees the uniform shear stress in different radii on substrate. Silicone membrane has been attached to bars that have the ability to move up and down to create this type of mechanical stretch movement. **b** Description of stress phase angle through cyclic shear and stretch stresses with normalized amplitudes. The time interval between rotating cone and oscillating jaws provides SPA

To generate and apply pulsatile shear and circumferential stresses separately, a special custom-made bioreactor was utilized as previously described. Briefly, the device consisted of a cone and plate mechanism by which shear stress was applied on the cell seeded plate. ASCs were seeded on a collagen coated (1 mg collagen/mL in 0.1% acetic acid, Sigma, USA) silicone membrane (Iran Polymer and Petrochemical Institute) and were exposed to cyclic shear stress of 0–2.5 dym/cm^2^. The magnitude of shear stress was achieved by a proper design of angle and dimensions of the cone, and considering culture medium as a Newtonian fluid with constant viscosity. Concurrently the membrane was subjected to 10% cyclic stretch through attachment to moving grippers (Fig. 1a). Two separate stepper motors (Delta electrics, Taiwan) provided the rotating movements of cone and oscillating movements of jaws. Shear stress has valued as high as 10–15 dyn/cm^2^ in some locations of vascular system near to the heart, but these locations generally experience in-phase shear stress and tensile stretches, and as a result, they are not theoretically vulnerable. However, the farther locations of the vascular system, which has been exposed to lower amount of shear stress, experience higher amounts of SPA, and therefore, they are more prone to some pathological conditions. Therefore, it has been tried to mimic these situations, and therefore, lower amount of pulsatile shear stresses have been utilized. A desired SPA was provided by calculated time delay between the rotation of cone and oscillation of jaws (Fig. 1b).

### Gene expression

For assessing the level of endothelial specific gene expression in ASCs among test and control samples, a real-time PCR assay was accomplished. Three independent experiments were performed in duplicate for real-time PCR. After application of mechanical forces on ASCs, the whole RNA was extracted by QIAGEN RNeasy plus mini kit (QIAGEN, USA) according to the manufacturer instructions and stored at − 80 °C. The optical density of the RNA was determined and 1000 ng of *RNA* was reversely transcripted to cDNA by QuantiTect, QIAGEN Reverse Transcription Kit (QIAGEN, USA).

The expressions of three endothelial specific genes which are essential in the function of ECs were analyzed among control and test samples. FLK-1 is an essential endothelial marker involved in permeability, migration, proliferation, apoptosis, and vasodilation. VE-cadherin is mostly known as an endothelial specific intracellular adhesion protein which contributes to vascular permeability and provides adhesive structure for inner layer of blood vessels and is necessary for expansion and branching of vessels. Since this marker is responsible for proper adhesion among endothelial junctions, alteration in its level may lead to passage of unwanted large molecules such as lipids, leading to atherogenesis (Guo and Hamilton 1996; Suhalim et al. 2012). On the other hand, vWF is generally activated in endothelial injury. This marker is expressed exclusively by mature ECs mostly during pathological conditions specially thrombosis and plays vital role in healing of endothelial injury through binding (Doggett et al. 2002).

SYBR green-based primers were designed by the Primer Express software (version 3) with the sequences as follows: house-keeping gene, glyceraldehyde 3-phosphate dehydrogenase (GAPDH), Forward, 5′-ACACCCACTCCTCCACCTTTG-3′ and reverse 5′-TCCACCACCCTGTTGCTGTAG-3′; FLK-1, forward, 5′-GACTTCCTGACCTTGGAGCATCT-3′ and reverse 5′-GATTTTAACCACGTTCTTCTCCGA-3′; vWF, forward, 5′-TCTGTGGATTCAGTGGATGCA-3′ and reverse 5′-CGTAGCGATCTCCAATTCCAA-3′; VE-cadherin, forward, 5′-TTTCCAGCAGCCTTTCTACCAC-3′ and reverse 5′-GGAAGAACTGGCCCTTGTCAC-3′; α-SMA, forward, 5′-ACACAACTGTGAATGTCCTGTGG-3′, and reverse, 5′-GGTAACGAGTCAGAGCTTTGGC-3′; SMMHC, forward, 5′-GAGACGAGAGGACATTCCACATC-3′, and reverse, 5′-TTGGAGAGGAAGGTGTAGTTGTTG-3′.

### Cell elastic modulus

To analyze elastic behavior of cell bodies atomic force microscopy (AFM) was used on cells after test duration for both test and control groups. Using Nanowizard 3 AFM device (JPK Instruments AG, Germany), the cantilever (CSC17/noAl, MikroMash), having nominal spring constant of 0.15 N/m, indented cells to apply 1 nN force. The spring constant of each cantilever was measured using a single force measurement test over a glass substrate and applying thermal noise method (Hutter and Bechhoefer 1993). For each point of indentation, the force-indentation depth curve was obtained and the modified Hertz theory was fitted to measure the Young’s modulus. According to tip shape of the used cantilever, the modified Hertz equation for conical shaped tips was implemented (Lin et al. 2007). In each test, 30 cells were randomly selected and indented in nearly 20 points over their nuclei.

### Actin fiber staining

Due to its established role in mechano-transduction and providing biomechanical strength for cells, actin fiber structure of cells was studied among test and control samples. Actin staining was performed by Phalloidin (Sigma, USA) and scanned by a confocal microscope. Briefly, the culture medium was removed and cells washed with PBS several times. Then formaldehyde (Sigma, USA) in 3.7% PBS was used for cell fixation followed by further washing. To make the membrane permeable to hue penetration, Triton X-100 0.1% (Sigma, USA) in PBS was added for 10 min. ASCs were washed again by PBS several times and 4 µg/mL phalloidin in PBS was added. The cells were then incubated at room temperature and protected from light for 45 min. After further washing, cells were transferred for confocal microscopy.

## Results

### Cell characterization

Flow cytometry assay revealed that more than 95% of cells expressed the mesenchymal stem cell specific markers (CD44 and CD90), while they did not express hematopoietic stem cell markers (CD45) (Table 1).

**Table 1:** Flow-cytometry results

M1 (Isotype)	M2		MSCs
99.05	1.02	CD44	+
14.61	84.5	CD90	+
11.44	88.2	CD45	−

M1 is isotype and M2 describes CD90, CD44, and CD45

### Multipotential assay

Results of multipotential assay demonstrated that ASCs were capable of osteogenic and adipogenic differentiations. Alizarin red staining described generation of the osteocyte cell matrix among ASCs exposed to osteogenic differentiating medium and adipose vacuoles were visible when ASCs were exposed to adipogenic differentiating medium.

### Gene expression

**Fig. 2:**
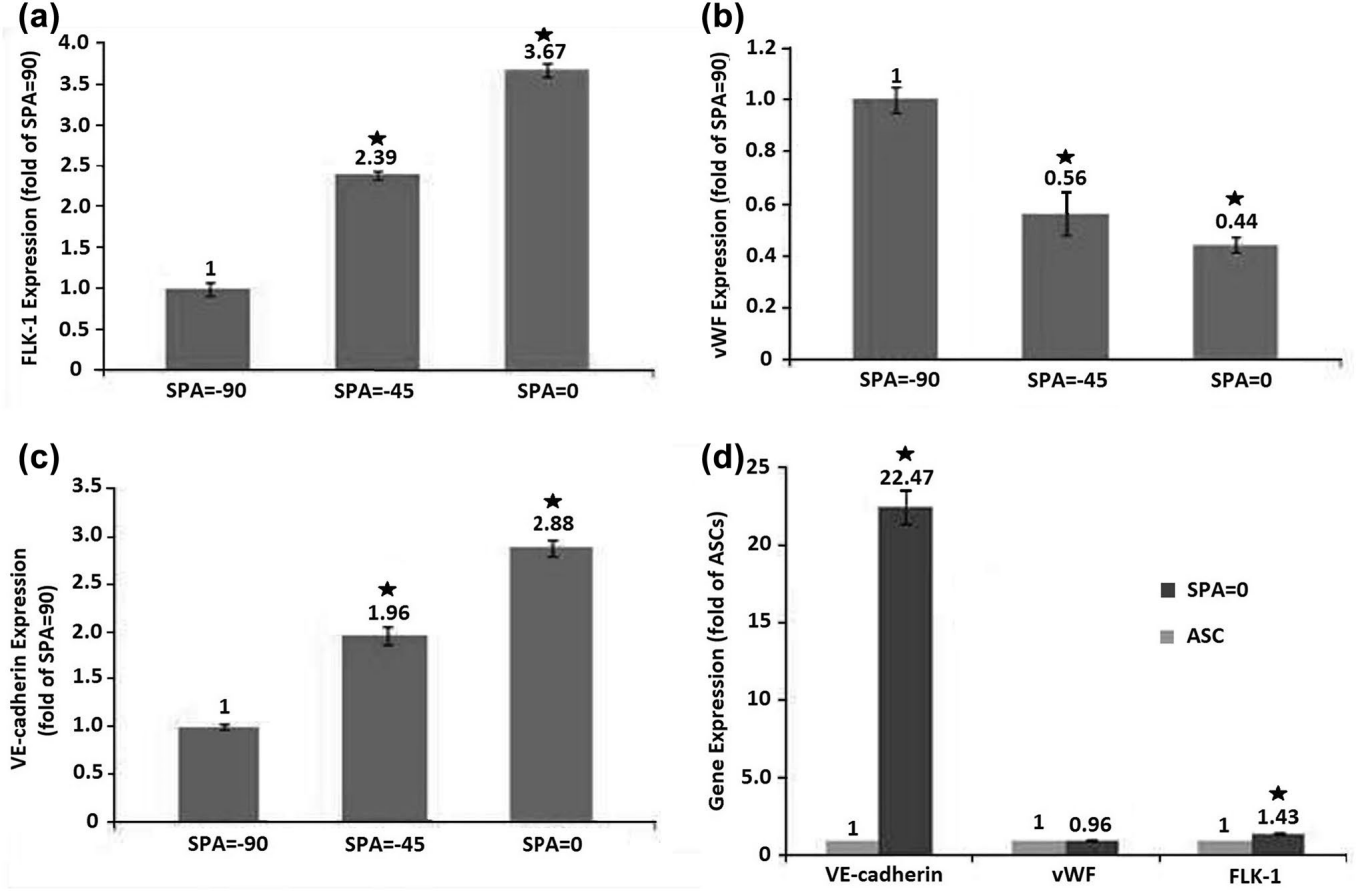
Expression of FLK-1 (**a**), vWF (**b**) and VE-cadherin (**c**) in ASCs, exposed to SPA = 0, − 45° and − 90°. **d** is for the comparison of SPA = 0 and ASCs as control group. The error bars indicate standard deviation and stars show statistical significant difference (*P* < 0.05). Elevation of SPA had negative effects on the expressions of endothelial specific genes in ASCs

Real-time PCR assay indicated altered gene expression in ASCs when exposed to cyclic stresses with differing SPA values. Figure 2 compares the expression of endothelial specific genes among test groups with SPA = 0, − 45°, − 90°, and control group. In general, the combined loading with differing SPA values caused significant elevation of FLK-1 and VE-cadherin among each test group compared to control group (*P* < 0.05). Application of combined cyclic stresses with SPA = 0 significantly elevated two major endothelial specific genes of FLK-1 and VE-cadherin by 1.43 and 22.47-folds (*P* < 0.05) (Fig. 2d). On the other hand, this loading regime did not cause any significant changes in the expression level of vWF (*P* > 0.05) (Fig. 2d).

Results demonstrated significant decrease of nearly 35 and 73% in the expression of FLK-1 when SPA reached − 45° and − 90° from 0°, respectively (*P* < 0.0.5). For VE-cadherin marker, such reductions were accordingly 32 and 65% (*P* < 0.0.5). The trend for vWF expression was reverse. When SPA reached the values of − 45° and − 90° from base line of 0, the expression levels of vWF increased by 44 and 127% correspondingly (*P* < 0.0.5). When alterations in the gene expression level were compared among all the test and control groups through ANOVA analysis, results indicated that regardless of increase or decrease, alterations were statistically significant for all three genes (*P* < 0.05).

### Cell elastic modulus

**Fig. 3:**
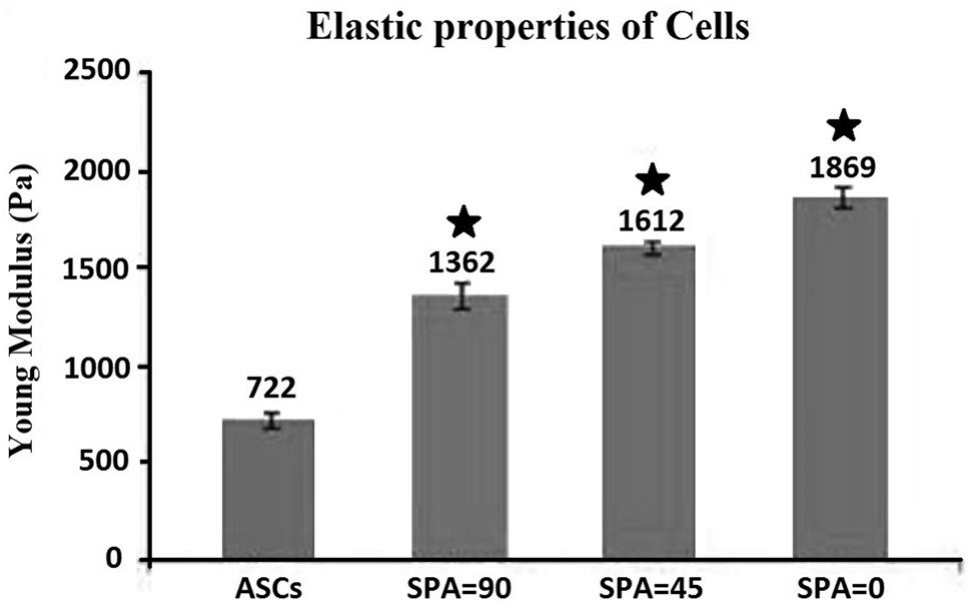
Young’s modulus of ASCs among control samples and samples exposed to combined tensile and shear stresses with SPA = 0, − 45°, and − 90°. Error bars show standard deviation and stars show significant difference when each test group was compared to control group (*P* < 0.05)

Alterations in Young’s modulus of ASCs when exposed to concurrent loadings with differing SPA are shown Fig. 3. The results indicated significant stiffening of cells for each test group compared to control samples (*P* < 0.05). The average increase of Young’s modulus of ASCs compared to that of control group was 159% for samples exposed to SPA = 0, 123% for samples subjected to SPA = − 45° and 89% among samples exposed to SPA = − 90° (Fig. 3). When all test and control groups were compared through ANOVA analysis, cell stiffening was found to be significant (*P* < 0.05).

### Actin fibers staining

**Fig. 4:**
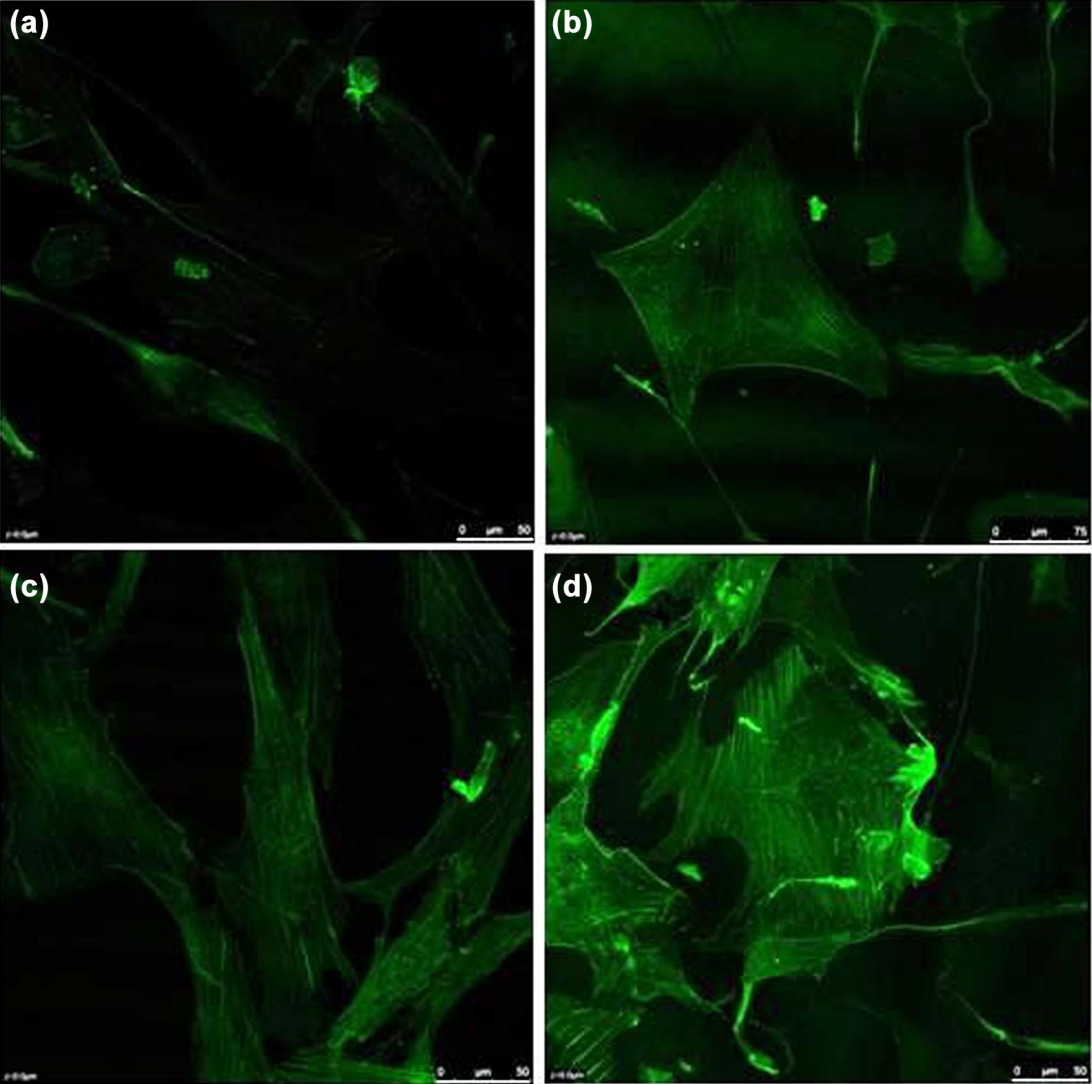
Confocal imaging of phalloidin staining of actin fibers for **a** ASCs without any biomechanical treatment, **b**–**d** ASCs exposed to combined cyclic shear and tensile stresses with SPA = − 90°, SPA = − 45° and SPA = 0, respectively

Phalloidin staining of actin fibers was carried out for test and control groups. Figure 4 shows samples of confocal microscope imaging of stained actin fibers among four groups. Results described that application of combined mechanical loadings had determinant effects on actin fiber structure and arrangement of cells describing more vivid and thicker fibers among samples treated mechanically in comparison to control samples. Compared to ASCs without any treatment, samples subjected to combined cyclic shear-tension developed distinct fibrous structure through stress fiber generation and organization (Fig. 4b–d). Among test groups, samples with SPA = 0 indicated the thickest fibers and highest generation of stress fibers (Fig. 4d). ASCs without any treatment (control samples) showed poor organized actin structure.

## Discussion

It is well established that biomechanical environment of ECs regulates endothelial vital function within the cardiovasculature (Li et al. 2005). Among arteries cyclic wall shear stress that is due to blood flow accompanied by cyclic circumferential stress caused by pulsatile blood pressure, as two main hemodynamic parameters, determine endothelial function and maintain the homeostatic state of arteries. A parameter range outside biological condition may cause endothelial injury which is the main source of human mortality (Tada et al. 2007). Moreover, the temporal phase angle between these pulses, has been emphasized to play highlighted role in endothelial behavior (Qiu and Tarbell 2000a). It has been hypothesized that the value of stress phase angel (SPA) is a determinant of endothelial behavior. Cyclic shear and circumferential stresses are often markedly out of phase in the vulnerable regions of plaque formation and atherosclerotic arteries (Qiu and Tarbell 2000a; Tada and Tarbell 2005).

Here, we hypothesized that SPA is an influential parameter in differentiation of mesenchymal stem cells toward endothelial phenotype. This might give clue in achieving functional engineered ECs for therapeutical applications and indicate insight into mechanisms by which endothelial injury occurs in pathological conditions. We conducted experiments that assessed effects of differing SPA on behavior of ASCs through analysis of gene expression, elastic properties of cells, and actin cytoskeleton structure.

Results indicated that application of out-of-phase SPA resulted in lesser expression of vital endothelial genes and softer cell body with a diverse actin fiber skeleton. In other words, more negative SPA values have lagged the endothelial differentiation of ASCs. Among three vital endothelial genes, the expression level of vWF was less prominent, that is the only marker among three markers which is expressed more during pathologic conditions dealt with endothelial injury. The results are consistent with the idea that higher absolute values of SPA are correlated with dysfunctioning ECs.

FLK1 and VE-cadherin are two major markers essential in endothelial behavior. They are not only regulators of biological function of ECs, but also play major roles in mechano-transduction of environmental stimuli among which hemodynamic features are determinants in functionality of ECs within vasculature. A marked upregulation of VE-cadherin (up to 22.47-folds for in-phase shear-tension waves) was observed when ASCs were subjected to concurrent cyclic shear-tensile loadings. This confirms that a micromechanical environment similar to that of functional ECs within cardiovascular system assists endothelial differentiation of ASCs. However, this depends on the phase difference between two mechanical stimuli. In addition to permeability, migration and proliferation of ECs (Holmes et al. 2007), FLK-1, as an endothelial specific marker, determines strain energy density of membrane which directly affects the opening of ion channels and eventually regulates cell permeability (Sachs 2010). In current study the expression of FLK-1 in samples with SPA = − 90° was only 27% of those of SPA = 0.

Compared to VE-cadherin, a modest, yet statistically significant upregulation of FLK-1 (up to 1.43-folds for in-phase shear-tension waves) was observed. It has been demonstrated that VE-cadherin actively contributes to the control of endothelial cohesion and intracellular junctions (Ohashi et al. 2007). The interaction of blood with the surrounding tissues is mainly regulated by the endothelium. The appropriate function of VE-cadherin and claudin-5 that are the key elements in adherence and tight endothelial junctions would be determinant of the barrier properties of endothelium to unwanted macromolecules which induce atherogenesis. In some serious pathological conditions among arteries, abnormal increase of vascular permeability has shown to be evident (Gavard 2009). The loss of VE-cadherin occurs due to the permeability inducing factors (PIFs) which dismantle VE-cadherin functionality by phosphorylation, internalization, and catenin dissociation (Gavard 2009). In current study, the expression level of VE-cadherin of samples exposed to SPA = − 90° was only 35% of those exposed to SPA = 0.

The function of Willebrand factor (vWF) is rather different from other two genes, since it is related to injury conditions. The vWF marker plays an important role in platelet adhesion along the wound sites within the arteries (Sadler 1998; Wu et al. 2008). Past studies have explained that the platelet aggregation and adhesion is regulated by release of vWF (Vischer 2006), and subsequently, the high level of vWF has been attributed indirectly to atherosclerosis and thrombogenesis (Lip and Blann 1997; Vischer 2006). Hence, it is not surprising that results of our research describes signified higher values of vWF as the SPA reaches higher negative values, since higher SPA values have been shown to be associated with atherosclerosis and thrombogenesis (Qiu and Tarbell 2000a). The average vWF level was 1.78 and 2.27 higher corresponding to test groups with SPA = − 45° and SPA = − 90° compared to samples with SPA = 0.

The differentiation of ASCs was accompanied by cytoskeletal development through enhancement of actin fiber structure and generation and bundling of new fibers which results in stiffening of the cell body as measured in current study. However, such effect was weakened when cells were treated by more negative SPA values. The arrangement and organization of cytoskeleton are the determinant factor in Young’s modulus of cell bodies. The elastic modulus of cells is related to the content and arrangement of cytoskeletal elements especially actin fibers. Since cell stiffness is influenced by the culture conditions and substrate properties (Byfield et al. 2009), a wide range of 2000–7000 Pa has been suggested for matured human aortic endothelial cells and human umbilical vein endothelial cells (Mathur et al. 2001). Our results suggest that ASCs treated by synchronic phase of shear and circumferential stresses (SPA = 0) has the closest value of elastic modulus to that of normal mature ECs. It has been well demonstrated that function of endothelial cells is related to the elastic property of the cell body and cytoskeletal arrangement (Mathur et al. 2001). In some clinical conditions, the stiffness of ECs was shown to be reduced (Lekka et al. 2012). Highly deformable ECs are more prone to passage of large molecules (especially in atherogenesis) and other cells through endothelium (Simoneau et al. 2012). Such phenomenon is also of great importance in cancer development in which cancerous cells pass through endothelial junctions during metastasis state (Simoneau et al. 2012).

Cell stiffness is determined by the thickness and arrangement of bundles of actin fibers. Application of biomechanical stimuli results in remodeling of the cell body mainly through alignment and generation of stress fibers (Wang et al. 2001). In this study, the effects of cyclic shear and circumferential stresses have been emphasized on both cell elasticity and actin structure. Cells under mechanical stimuli were shown to be stiffer and have more actin content than ASCs (Figs. 3, 4). Moreover, the phase difference between shear and tensile stresses was shown to be significantly effective. When the phase difference was smaller, the cell body was stiffer and the actin structure became richer. It is likely that application of smaller phase difference on ASCs during differentiation results in cells with characteristics more similar to functional ECs. While in all biomechanically motivated groups, actin fibers were semi-organized suggested to be in the direction of minimal substrate deformation (Wang et al. 2001), the fibers in group of SPA = 0 were shown to be thicker and more organized compared to other groups of SPA = − 90° and SPA = − 45° (Fig. 4). It can be implied that cell stiffening was mostly due to enhanced cytoskeleton. The shear stress or tensile stretch alone cannot perfectly and completely mimic the inherent unique mechanical environment of endothelial cells and this fact is even more highlighted when we understand that shear stress or tensile stretch alone have been used on other cell phenotypes as a motivator for differentiation of mesenchymal stem cells. For instance, the uniaxial and equiaxial tensile stretches alone have been widely used for osteogenic (Qi et al. 2008) and cardiomyocyte (Huang et al. 2012) differentiation of stem cells and our investigations showed that not only for endothelial differentiation, but also shear stress alone has been utilized for chondrogenic (Alves da Silva et al. 2011) and osteogenic (Yourek et al. 2010) differentiation of different colonies of mesenchymal stem cells. The idea behind these applications is that, for example, shear stress alone does exist in many in vivo biological environments such as articular joints, as the synovial fluid flows during movement, and therefore, shear stress alone can induce chondrogenic differentiation or a shear stress that is created in trabecular bone which performs as a stimulator of osteoblasts and osteoclasts. Even shear stress alone has been used for cardiomyogenic differentiation (Huang et al. 2010). However, the combination of shear stress and tensile stretch can be regarded as a unique mechanical environment special for blood vessels and logically their results have more relative significance.

## Conclusion

Beside the independent role of biomechanical forces on cell behavior, their interaction may also influence cell behavior. In this study, the endothelial differentiation of ASCs was shown to be influenced by shear/tensile stresses and their interaction characterized by SPA. In was shown that application of in-phase SPA incites conditions related to healthy ECs, while larger phase differences were related to dysfunctioning ECs. This may provide some clues for determination of pathophysiological mechanism that lies under endothelial injury and atherogenesis. In addition, the results assist in determination of proper mechanical stimuli for functional endothelial differentiation of mesenchymal stem cells.
